# Extending the Shelf Life of Strawberries: Physicochemical and Antibacterial Effects of Carboxymethyl Cellulose and Gelatin Coatings With Lemon Essential Oil

**DOI:** 10.1002/fsn3.70222

**Published:** 2025-04-30

**Authors:** Arkan Mohammed Hassan, Ammar B. Altemimi, Babak Ghanbarzadeh, Perihan Adun, Khaled Arab, Sonya Ibrahim, Farhang Hameed Awlqadr, Mohammad Ali Hesarinejad, Tarek Gamal Abedelmaksoud

**Affiliations:** ^1^ Faculty of Science, Department of Chemistry University of Garmian Iraq; ^2^ Food Science Department College of Agriculture, University of Basrah Iraq; ^3^ College of Medicine, University of Warith Al‐Anbiyaa Karbala Iraq; ^4^ Faculty of Agriculture, Department of Food Science and Technology University of Tabriz Iran; ^5^ Faculty of Engineering, Department of Food Engineering Near East University Mersin Turkey; ^6^ Food Science and Quality Control Halabja Technical College, Sulaimani Polytechnic University Sulaymaniyah ‐ Iraq; ^7^ Department of Food Sensory and Cognitive Science Research Institute of Food Science and Technology (RIFST) Mashhad Iran; ^8^ Faculty of Agriculture, Food Science Department Cairo University Giza Egypt

**Keywords:** Carboxymethyl cellulose, edible coating, gelatin, lemon essential oil, strawberry

## Abstract

Edible coatings are a thin layer of substances that are put on the surface of food. This work was designed to investigate strawberry coating prepared of carboxymethyl cellulose (CMC), gelatin (G) enriched with lemon essential oil (LEO) in various concentrations (0.5%, 1.5%, 3%), on the antimicrobial characteristics, shelf life, physicochemical, and sensory properties of strawberries preserved for 16 days at 4°C ± 1°C and an RH of 85% ± 5%. It was found that adding LEO to the CMC + G coating inhibited yeast and mold growth as well as decreased weight loss. The total flavonoid (TF), total phenol content (TPC), ascorbic acid, and antioxidant activity (AOA) all decreased slowly. Furthermore, the CMC + G + LEO combination reduced fruit deterioration due to respiration‐related cell wall degradation and delayed titratable acidity (TA); pH changes, as well as losses of the fruit's total soluble solid (SS), were decreased. Likewise, the simultaneous use of CMC, G, and LEO in the sensory assessment (texture, flavor, appearance, and over all acceptance) improved aroma and appearance in the sensory assessment of the current research employing CMC + G + LEO 3%. It also proved to be efficient in reducing firmness loss, total flavonoids, ascorbic acid, TPC, and AOA in strawberry fruits compared with the uncoated.

## Graphical abstract

CMC + G + LEO coatings inhibited microbial growth and extended strawberry freshness for 16 days at 4°C. Coated strawberries showed reduced weight loss, better firmness, and stable physicochemical properties (soluble solids, acidity, pH). The 3% LEO coating maintained higher antioxidant levels, including ascorbic acid, phenols, and flavonoids. Sensory tests showed improved aroma, taste, and acceptability with CMC + G + LEO coatings compared with uncoated fruit.

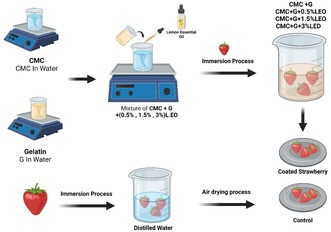

## 1 Introduction

Strawberries are in high demand due to their versatile applications in the processing industry and fresh consumption, especially in confectionery and preserves. These fruits are not only known for their delightful aroma and taste but are also rich in nutrients such as antioxidants, fiber, vitamins, and minerals (Khoshdouni Farahani et al. 2025). Moreover, strawberries contain a substantial amount of phenolic compounds, flavonoids, and anthocyanins, which have been linked to a reduced risk of cardiovascular diseases and cancer (Hjartåker et al. 2015; Oyebode et al. 2014). However, strawberries, like many perishable foodstuffs, gradually lose their appearance, nutritional value, and flavor during the long processes of storage, handling, and transportation. Without proper protection, damage can occur, even if it is not immediately visible, within a few hours or days (García et al. 2009). The research is primarily concerned with the packaging of food using natural biopolymers, with an emphasis on food safety and extending shelf life. In particular, the study investigates biodegradable materials with antimicrobial properties in coatings or films that are not associated with health concerns. These polymers offer advantages by reducing the need for artificial antimicrobial substances during food processing (Kumar et al. 2020). Biodegradable coatings and films can also serve as carriers for active substances like antimicrobials and antioxidants, thereby enhancing food health and nutrition (Kaur et al. 2024). Among the promising materials is carboxymethyl cellulose (CMC), a nontoxic, water‐soluble anionic polymer derived from cellulose. CMC is both affordable and widely available (Abdullah et al. 2023). CMC films and coatings exhibit excellent flexibility and transparency, and their properties are enhanced when combined with other polymers such as kefiran or starch (Hasheminya et al. 2019; Sanchez‐Gonzalez et al. 2011). Furthermore, CMC is commonly utilized for blending with gelatin because of its exceptional biocompatibility and widespread accessibility (Hasheminya et al. 2019). CMC coatings or films exhibit excellent barrier properties against carbon dioxide (CO_2_), oxygen (O_2_), fats, and oils because of their densely packed network. However, they have limited resistance to water vapor. The resistance of cellulose derivatives exhibits excellent gas impermeability when used alone, but when used in conjunction with plastics, this considerably reduces gas barrier characteristics (Ghanbarzadeh et al. 2011; Paunonen 2013).

Gelatin (G) is another well‐studied water‐soluble protein known for its functional properties such as stabilizing, thickening, emulsifying, gelling, and film‐forming abilities (Etxabide et al. 2017; Nazmi et al. 2017). Moreover, gelatin‐based coating when mixed with CMC can act as a barrier against oxygen, carbon dioxide, and fat, thus preserving food from dehydration, light, and oxygen exposure (Zhang et al. 2021; Azarifar et al. 2019). The combination can overcome the limitation of pure gelatin‐based films, enhancing their functional performance (Ali et al. 2025).

Essential oils (EOs) are utilized to optimize the efficiency of biodegradable packaging due to their fundamental properties, including antioxidant, antimicrobial, and hydrophobic properties (Xylia et al. 2021). EOs are plant‐derived compounds subject to the limited water vapor for edible film and coating (Xylia et al. 2022). The EOs contain terpenoids and terpenes components, as well as act as antibiotics, consumer desire has increased as a result of its widespread use to superior quality and keep food safe. In addition, gelatin incorporated with *Mentha pulegium* essential oil coating could function as bioactive packaging to enhance shelf life and provide an alternative to pesticide application (Aitboulahsen et al. 2018). According to Sogvar et al. (2016) ascorbic acid was incorporated into *aloe vera* coating to enhance the quality of strawberries throughout the preservation period. The previous study by Dong and Wang (2017) utilized a combination of CMC and garlic EO to create composite coatings. The coatings assist in protecting the nutritional composition of strawberries by delaying weight loss and senescence. A composite edible coating made of chitosan, lemongrass essential oil, and thyme essential oil was developed and applied to strawberries in a previous study by Ibrahim et al. (2017). The coated strawberries were stored at 4°C and assessed over 15 days. The results demonstrated that the coating effectively prolonged the shelf life of the strawberries. Additionally, it suppressed microbial growth, preserved anthocyanin content, and reduced weight loss. In a recent investigation by Dashipour et al. (2015) (Xylia et al. 2021), edible coatings including pectin, cellulose nanocrystals, glycerol, and lemongrass EO were employed (Asif et al. 2023). Utilizing these coatings has demonstrated a significant outcome in protecting the quality of strawberries. The study aims to assess the effectiveness of a coating comprising carboxymethyl cellulose (CMC), gelatin (G), and in combination with lemon EO prepared on the shelf life and quality, as well as how different concentrations of LEO affect the physicochemical characteristics of the strawberries during storage at 4°C.

## 2 Materials and Methods

### 2.1 Materials

The parous strawberry fruit was bought freshly on the farm from Tehran, Iran, and was directly transported in a package container under controlled conditions to the laboratory. Strawberry uniforms were sorted based on size, shape, and color (80% red, 20% yellow and white) with no indications of fungal spoilage, physical injury, and blemishes. practical grade of CMC was obtained from (Shandong, china). and G (220 bloom) supplied from Tabriz, Iran. LEO came from Esfahan's Tabib Daru. Tween (80) and glycerol were acquired from Merck. The following materials were purchased from Sigma: DCIP, Folin‐Ciocalteu, 0.1 N Phenolphthalein, NaOH, DPPH, metaphosphoric acid, and potato dextrose agar (PDA). Key parameters evaluated by the samples include weight loss, pH, titratable acidity, firmness, total flavonoids, ascorbic acid, antioxidant activity, total phenols, mold and yeast counts, and sensory attributes.

### 2.2 Coating Formulation

The coating solution was prepared with a few adjustments during the preparation process, according to Dashipour et al. (2015). The mixture was prepared from CMC (1 g in 70 mL distilled water agitated at 70°C for 55 ± 5 min). Then the solution was mixed with gelatin (0.5 g in 30 mL of distilled water at 40°C for 25 min) for 25 min and then put in the water bath for 40 min. To enhance stability, 0.675 mL of the glycerol‐based composite coating was stirred at 25°C continuously for 10 min. LEO (0.5%, 1.5%, and 3% v/v) based on composite coating was incorporated, and the solution was stirred at room temperature for 30 min to enhance the antioxidant compounds and quality. Surfactant Tween 80 (1% v/v according to LEO) was added to CMC + G, which incorporated LEO. Ultimately, the prepared solution was agitated at 20°C for 120 min.

### 2.3 Application of Edible Coating on Strawberries

Strawberries were cleaned by soaking them for 2 min in distilled water containing 2% sodium hypochlorite, washed three times with distilled water, then air dried at room temperature. Strawberries were coated using the immersion method. Two samples were created: (1) control, which consisted of fresh strawberries without any solution, and (2) immersed in coatings: Samples were submerged in the formal solution for 45 s, after which they were allowed to drip off on stainless‐steel screens (1.1 cm × 1.1 cm). The solution was drained and left to air dry at room temperature for 2 h. The labels applied to the strawberries were CMC + G, CMC + G + LEO 0.5%, CMC + G + LEO 1.5%, and CMC + G + LEO 3%. Lastly, four strawberries were kept in polypropylene boxes for 16 days at 4°C ± 0.5°C and 85% ± 5% RH, with three duplicates kept in the refrigerator.

### 2.4 Physicochemical Properties of Coated Strawberry

#### 2.4.1 Weight Loss

The experiment involves regularly measuring the weight of both coated and uncoated strawberries stored at 4°C over a period of 16 days. This procedure aimed to assess the extent of weight loss during storage. The outcome was quantified as the percentage reduction in the original overall weight. Each group's weight reduction was measured in three replicates with six fruits.

#### 2.4.2 Firmness of the Strawberry Coating

The firmness was examined and assessed for uncoated and coated strawberries with a Testing Machine texture analyzer SANAF Instruments Model SUT‐50KN, Iran. We found the penetration force (N) using a stainless‐steel cylinder probe with a 4 mm thickness and a constant rate of 5 mm per s^−1^ at the sample's surface. As reported by (Avalos‐Llano et al. 2018) two samples were obtained from the center zone of each strawberry on the opposite side, using three duplications.

#### 2.4.3 The Measurement of pH, Titrable Acidity (TA), and Total Soluble Solid (TSS)

The strawberries were chopped and then homogenized at 15000 rpm for 2 min. A pH meter (Oakton PH 550 Benchtop, Germany) was used to assess the pH at room temperature (Saleem et al. 2021). After filtering through cheesecloth, a hand Refractometer (Model N‐3000E, Atago, Japan) was used for measuring the TSS of strawberries at 20°C. Strawberries were tested for acidity using the titration technique. For 30 min, 5 g of prepared strawberries were soaked in 45 mL of hot distilled water (80°C). After the aliquot was filtered through cheesecloth, A phenolphthalein indicator of 3 drops was added, and a persistent pale pink color appeared (Velickova et al. 2013). To determine the total acidity in the diluted puree relative to citric acid by applying this formula (Equation 1):

(1) $$\mathrm{TA}=\frac{V\left(\text{NaOH}\right)*0.1*0.064}{m\;\text{aliquote}}\times 100$$

Where: V (NaOH) is the volume of titrated NaOH in mL of a standardized 0.1 N NaOH solution. 0.064 is the citric acid conversion factor. M aliquot: is the mass of the material used in the titration.

#### 2.4.4 Mesurement of Ascorbic Acid

Nair et al. (2018) report that the titration technique was used to quantify the ratio of vitamin C in strawberries using 2, 6‐dichlorophenol‐indophenol (DCPIP) dye. Ascorbic acid was extracted from 10 g of strawberries crushed with a mortar and pestle and 100 mL of 3% (HPO_3_) metaphosphoric acid. After centrifugation at 4°C for 15 min at 10000 rpm, the mixture was filtered using Whatman No. 1 filter paper. 10 mL from the filtrate was then titrated until the rose‐pink color remained for 15 s. Ascorbic acid levels were given in mg per 100 g of fresh fruit.

#### 2.4.5 Methanolic Extract Preparation for Coated Strawberries

Homogenized 1 g of fresh strawberries was used in a chopper (IKA Model T 25 Digital, Germany) at 11,000 rpm and 4°C for 3 min. According to Avalos‐Llano et al. (Xylia et al. 2022), but with a few adjustments, 16 mL of 80% methanol (8:2 v:v H_2_O) was agitated for 1 h with specimens and then placed in an ultrasonic bath for 10 min. Finally, at 4°C, the combination was centrifuged (AVANTI J‐26 XP Beckman Coulter Inc., Fullerton, CA, USA) at 9000 rpm for 14 min, and Whatman paper No. 4 was used to filter.

#### 2.4.6 Total Phenols Content

The total phenol has been determined through UV spectrophotometry, with the Folin–Ciocalteu oxidizing agent. Following the procedure from (Škerget et al. 2005) with certain changes, for 1 mL of the extracted sample, 2.5 mL of Folin‐Ciocalteu was used. 2 mL (0.75%) of Na_2_CO_3_ was added after 4 min. Following 1 h of incubating the prepared mixture, the absorbance was identified at 760 nm. The outcome was expressed as the amount of gallic acid equivalent (mg GAE/100 g‐1 extract).

#### 2.4.7 Total Amount of Flavonoids

An aluminum chloride colorimetric test was performed with 1 mL of extracted strawberry samples to measure the total flavonoid content (Zhishen et al. 1999). 4 mL of 80% methanol was put in a 10 mL volumetric flask, and the specimen was poured into it. Then, 0.3 mL of NaNO_2_ was diluted tenfold in distilled water, and 0.3 mL of 10% AlCl_3_ diluted tenfold in 80% methanol was added to the same flask. Following a 6‐min incubation, 2 mL of 1 M NaOH was added, and finally, the flask was filled with 10 mL of 80% methanol, which was then incubated for 1 hour. The absorbance at 510 nm was then computed. The total flavonoid content in triplicate samples was expressed as mg of catechin equivalents (CE)/100 g^−1^ based on fresh weight.

#### 2.4.8 Antioxidant Activity

According to (Pineli et al. 2011), strawberry extracts' antioxidant activity (AOA) was assessed. An aliquot of 0.31 mL (DPPH, 3.5 mg/100 mL of 80% methanol) with 0.1 mL of extract was mixed. the absorbance at 517 nm was determined by dark incubation at 25°C for 30 min. Trolox equivalents (μmol TE g‐1 dw) were used to express the final result. The formula of AOA Equation (2):

(2) %inhibition=A0−A1/A0×100

#### 2.4.9 Analysis of Mold and Yeast

The microbiological tests were reported by Shahbazi (Saleem et al. 2021) and carried out with a few modifications to determine the total yeast and mold. 1 g of strawberries that had been chopped into little pieces was carefully placed into a sterilized tube holding 9 mL (0.9% sterile saline solution). The mixture was spread out across the potato dextrose agar (PDA) surface, a selective medium, after being homogenized (IKA Model T 25 Digital, Germany) for 2 min at 9000 rpm. Serial dilutions of strawberries (10^−1^, 10^−2^, and 10^−3^) were incubated on flipped plates at 25°C ± 1°C for 5 days. A count of the colonies was conducted after 3 and 5 days, and the number of mold and yeast findings was expressed per gram of fresh weight.

#### 2.4.10 Sensory Analysis

Following the first day's coating application, the strawberry fruits were evaluated in the sensory laboratory. Fresh strawberry (control). Samples were distributed in a fully randomized way to participants at a temperature of 20°C ± 1°C, with 5‐digit random numbers on white plates. Nine evaluators graded the sample odor, texture, color, taste, and overall acceptability (Azarakhsh et al. 2014). Four female and five male food engineers, ranging in age from 25 to 44, made up the board of skilled panelists. The characteristics were scored on a 10‐point hedonic scale, with (1) indicating insufficient color, nasty texture, undesirable aroma, texture lacks aftertaste, and no acceptance, while (10) indicated a flavor that is similar to perfect, a brilliant color, great overall acceptability, excellent texture, and powerful aftertaste.

#### 2.4.11 Statistical Analysis

A randomized design was employed for the experiment. Data variations were analyzed for significance using Duncan's multiple range test and one‐way (ANOVA). Differences were considered statistically significant at a *p* value of < 0.05 level. All statistical analyses were performed using SPSS software.

## 3 Results and Discussion

### 3.1 Weight Loss in Strawberry

**FIGURE 1:**
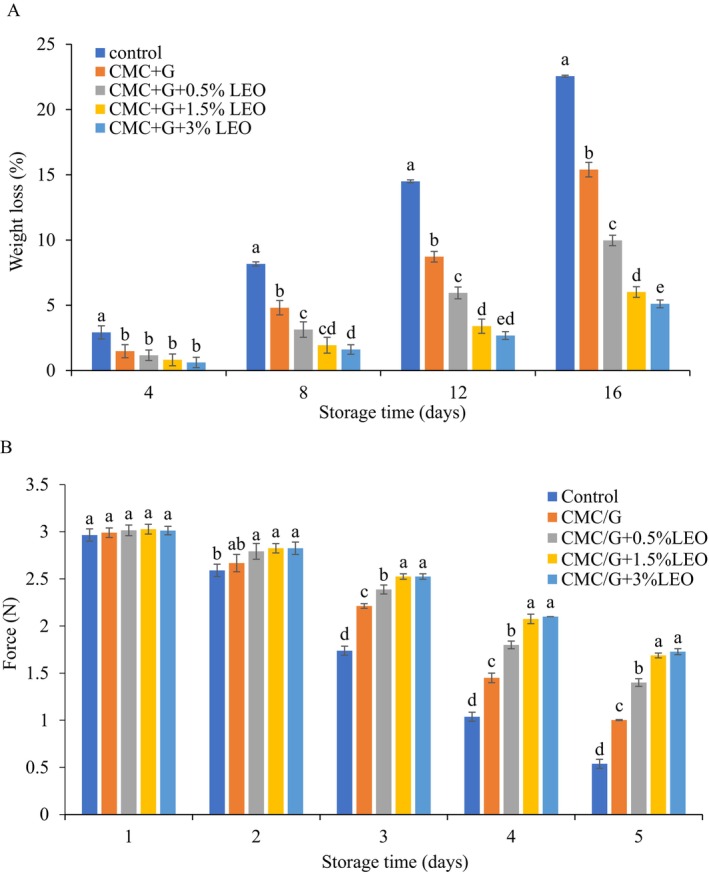
Effect of a combination of CMC + G with LEO on the (A) weight loss and (B) firmness of strawberries for storage in day at 4°C.

Weight loss in strawberries was monitored during storage at 4°C ± 0.5°C under different coating treatments (Figure 1A). The study revealed that as the storage duration increased, the rate of weight loss also increased. However, coated strawberries experienced significantly less weight loss compared to uncoated samples. The minimum weight loss of 5.10 g was observed in strawberries coated with CMC + G + 3% LEO while the uncoated strawberries exhibited the maximum weight loss of 22.55 g. The CMC + G + LEO coating that developed on the coated fruits' surface slowed the moisture migration resulting in weight loss and shrinkage. There was a significant difference (*p* < 0.05) in the weight loss, CMC + G coated samples exhibiting greater loss of weight results in contrast with CMC + G + LEO. The hydrophilic structure of the CMC can be reduced by LEO. Additionally, the interaction between the low concentration of LEO and the CMC developed an effect of binding. This may minimize the possibility of hydroxyl groups interacting with water molecules, thereby combination coating led to improving water resistance. Moreover, (Shahbazi 2018; Gol et al. 2013) also discussed the coating technique, such as (CMC) and chitosan (CH) containing *Mentha spicata* EO (MSO 0.1% and 0.2%) coatings, and then (CMC), hydroxypropylmethyl cellulose (HPMC) and composites with chitosan (CH) coatings, which prevented fruits from losing weight during 12 days.

### 3.2 Firmness of the Coated Strawberries

It is well known that when strawberries naturally lose water, their cell walls weaken and alter in texture over time. Figure 1B shows that coated and uncoated strawberry firmness decreases at 4°C ± 0.5°C after 16 days of storage. Furthermore, regardless of treatment, fruit hardness decreases during storage. However, CMC + G + LEO (3% and 1.5%) efficiently kept the firmness characteristic of strawberries, which softened gradually (3.01 N to 1.72 N and 3.02 N to 1.68 N), When compared to (CMC + G + LEO 0.5%, CMC + G and control) as (3.01 N to 1.4 N, 2.99 N to 1.0 N and 2.96 N to 0.53 N). Studies by (Velickova et al. 2013; Perkins‐Veazie 2010) indicate that parameters such as cell‐to‐cell contact, cellular turgor, and cell wall strength influence strawberry firmness. One important aspect that might be specified as influencing customers' judgments regarding the products is firmness. In addition, fruit softening reduces both the quality of fruit and customer demand. According to (Bhaskara Reddy et al. 2000), coatings or fresh strawberries lose some of their softness when they are kept in a cooled environment. The reduction in water vapor permeability caused by LEO enhances the performance of CMC + G films in preserving fruit during cold storage, leading to a noticeable reduction in fruit softening. Additionally, the metabolic activity will be decreased during the entire process.

### 3.3 Chemical Analysis

#### 3.3.1 Total Soluble Solids (TSS) of Coated Strawberries

**FIGURE 2:**
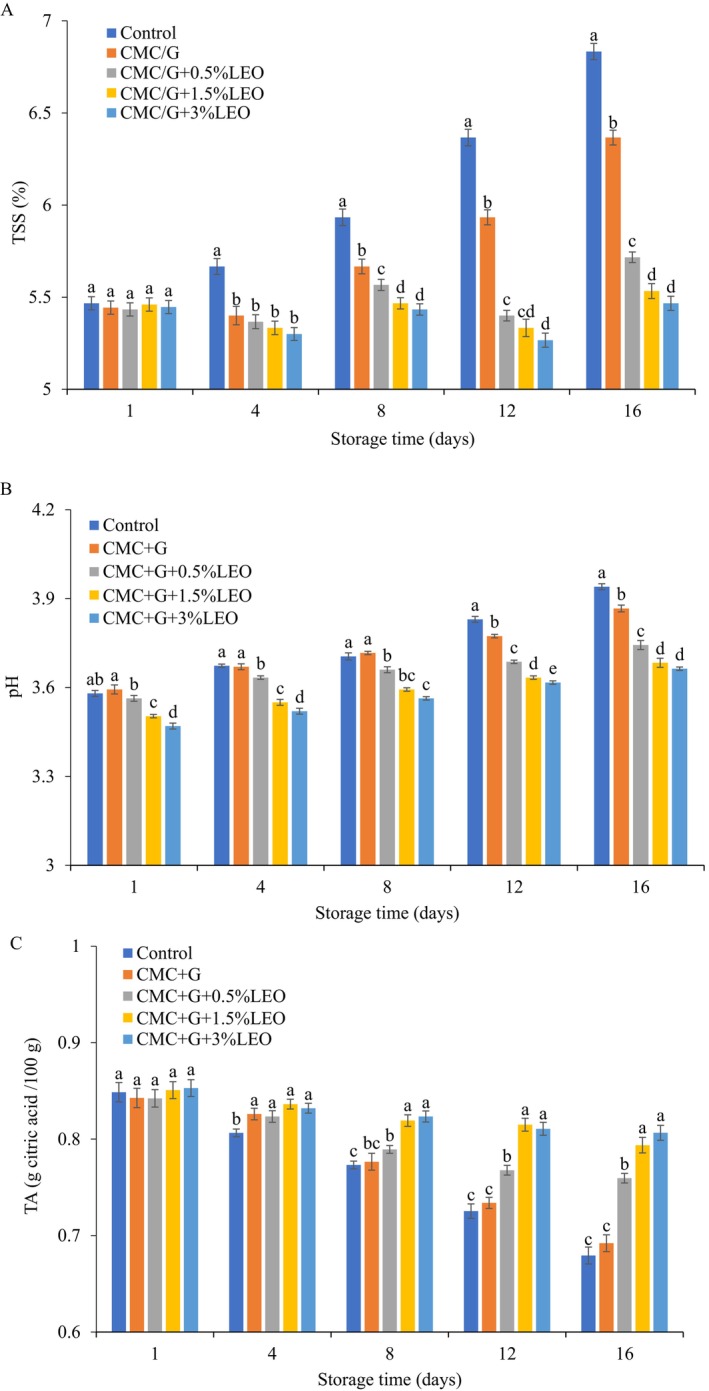
Effects of coated strawberry on the (A) soluble solid content (SSC), (B) pH change, and (C) titration acidity during 16‐day storage at 4°C.

According to this experiment, the increase in TSS is attributed to the breakdown of fruit polysaccharides into soluble sugars during storage. The strawberries coated and uncoated TSS levels were increased during storage at 4°C ± 0.5°C, as displayed in Figure 2A. Furthermore, rising TSS content in fruit coating was further studied by (Hernández‐Muñoz et al. 2008; Rivera‐López et al. 2005), indicating that the increase in soluble solids in strawberries results from starch conversion to soluble sugars in the cell wall. comparing the TSS among all the treatments, TSS increased to 6.83% in uncoated fruits, as compared with fruits coated with CMC + G or CMC + G + LEO. The sample coated with a higher concentration of LEO (3%) was observed to have a slower increase in TSS, reaching 5.46% when compared to the CMC + G 6.4% sample. likewise, as suggested by (Perdones et al. 2012) research, the fruit's metabolic and senescence processes may be affected by the interactions between essential oils and cell membranes, which could cause an increase in sugar concentration of fruits and lower decay percentage.

#### 3.3.2 The pH of the Coated Strawberry

The effect of pH on uncoated and coated strawberries is displayed in Figure 2B. After 16 days of storage, significant differences were observed in pH values across all treatments: CMC + G + 3% LEO (3.66), CMC + G + 1.5% LEO (3.68), CMC + G + 0.5% LEO (3.74), CMC + G (3.86), and the control (3.94). These findings align with Shahbazi (2018), who found that *Mentha spicata* essential oil (EO) enhanced the preservation of CMC‐coated strawberries, reducing pH changes compared to uncoated samples. Our finding was close to the result reported by (Maftoonazad et al. 2008); the untreated samples exhibited a higher pH value ranged from 3.90 to 4.55, in contrast to the peaches coated with methyl cellulose 3.9 to 4.25, stored in the refrigerator for 15 days. Furthermore, this laboratory investigation's findings showed that CMC + G + LEO coating was superior to CMC + G coating in terms of pH retention. According to (Mannozzi et al. 2017), the increase in pH during cold storage is attributed to microbial activity and metabolic processes that alter organic acid composition.

#### 3.3.3 Titratable Acidity (TA) of Coated Strawberry

The TA values of strawberries uncoated and coated declined at 4°C when kept for 16 days, displayed in Figure 2C. At the end of storage, the values of the CMC + G coated and control strawberries were (0.69 g/100 and 0.67 g/100), respectively, and CMC + G + LEO (0.5%, 1.5% 3%) were (0.75 g/100 g, 0.80 g/100 g, 0.79 g/100 g). Whereas the coatings using CMC + G combined with LEO exhibited their highest levels. CMC + G + LEO (1.5%, 3%) coated strawberries gradually reduced TA. According to (Wani et al. 2021), the edible coating's gum Arabic, xanthan and carrageenan coatings containing antimicrobial agent on postharvest quality of strawberry effect on strawberry TA caused it to reduce slowly (1.45 to 0.79, 1.44 to 0.82, and 1.46 to 0.77) during preservation for 12 days, whereas control fruit exhibited a greater decline from 1.45 to 0.74. This may arise as a consequence of fruit gradually ripening or because the respiratory process uses organic acids, which accelerates the senescence process. (Khalifa et al. 2016) Expressed that chitosan film with olive oil might have an impact on two distinct important aspects of strawberries, including modified respiration and metabolic patterns slightly reduced. According to (Ali et al. 2019), applying an *aloe vera* gel coated likely decreased fruit senescence and oxidation, which preserved greater TA amounts in coated litchi fruit. Also, due to the fruit's rate of respiration, organic acid transforms into sugar decreased (Fagundes et al. 2015).

### 3.4 The Impact of Coating on the Quantity of Ascorbic Acid in Strawberries

**FIGURE 3:**
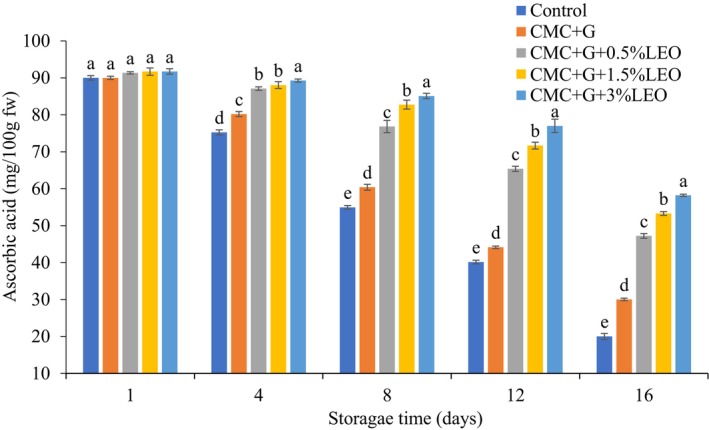
Effect of coating on ascorbic acid content of strawberry.

Figure 3 shows that coating slowly reduces the ascorbic acid content of strawberries at 4°C ± 0.5°C during 16‐day storage, compared to uncoated fruits. All treatments of coated strawberry, CMC + G + LEO (5%, 15%, and 3%) and CMC + G, differed significantly from one another (47.23, 53.31, 58.20, and 30.02) mg/100 g fw. though, ascorbic acid levels in uncoated fruits were shown to have decreased the most at the end of the shelf life, approximately 20.02 mg/100 g fw. In contrast, coatings using CMC + G + LEO (3%) showed the lowest decrease, at 58.20 mg/100 g. In a previous study (Dong and Wang 2017), a decrease in the quantity of ascorbic acid degradation was observed when strawberries were preserved using CMC enhanced with garlic essential oil for 6 days at 20°C. This could occur because oxygen diffusion is slowed down, which inhibits the enzymatic processes that cause ascorbic acid to be oxidized in fruits. According to the study Amal et al. (2010), the barrier characteristics were enhanced by adding essential oil to the biopolymer, which slows the deterioration of ascorbic acid by oxidative reduction.

### 3.5 The Impact of Coating on the Total Phenolic Content (TPC) of Coated Strawberries

**FIGURE 4:**
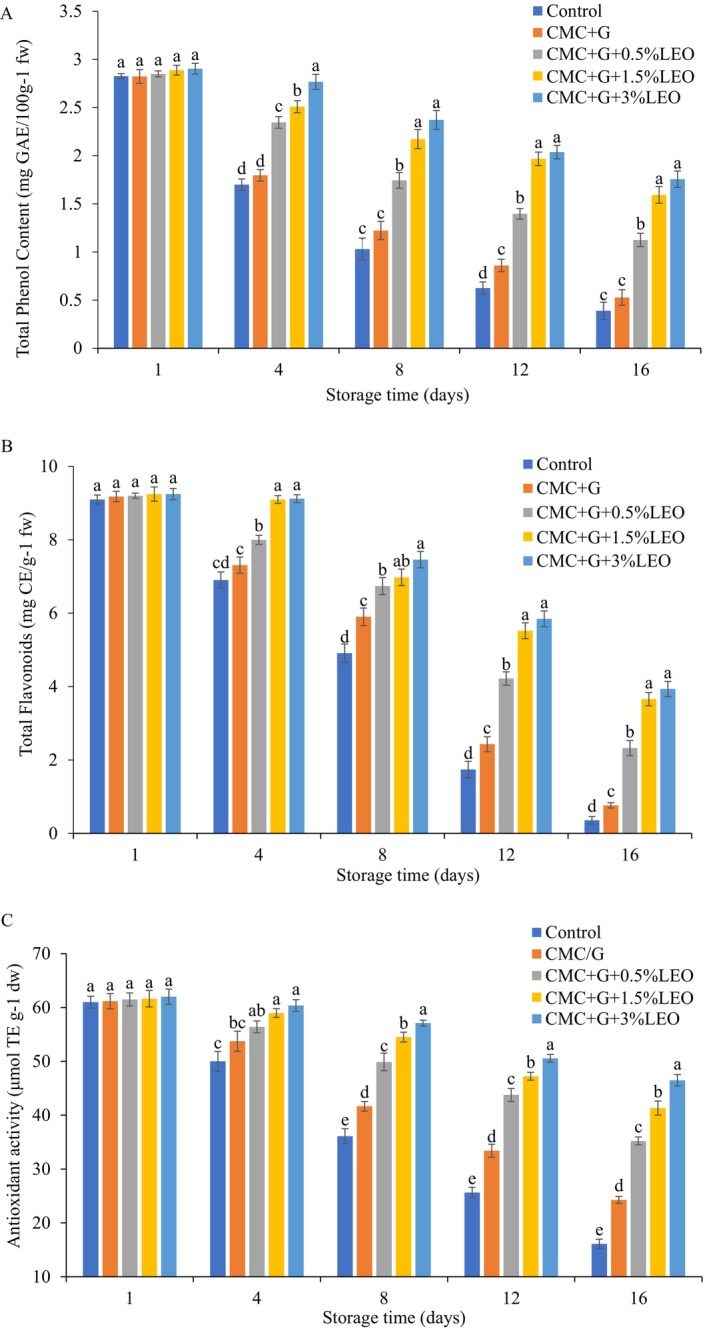
(A) Total phenol content, (B) total flavonoid content, (C) and antioxidant activity of strawberries during 16‐day storage at 4°C.

The fruit TPC reduced in uncoated and coated, demonstrated in Figure 4A. The TPC reduction was seen in the strawberries treated with CMC + G and CMC + G + LEO (varying concentrations), when compared to uncoated strawberries. Minimal reduction was observed in CMC + G + LEO (3%), at around 1.75 mg GAE/100 g. On the other hand, uncoated strawberries observed the greatest decrease in TPC, reaching 0.388 mg GAE/100 g during the shelf life of 16 days. (Gol et al. 2013) have discovered that fruit TPC decrease is caused by the disintegration of cell structure, which releases phenolics that are then affected by enzymatic oxidation. According to this study's findings (Saleem et al. 2021), chitosan with ascorbic acid combined significantly decreased phenolic loss and may benefit from this combination suitable for strawberries kept in the cold. This may be because less oxygen was available. According to (Wang and Gao 2013), TPC in coated fruit enriched with LEO forms a thin layer that is efficient for reducing respiration rate.

### 3.6 Impact of Coating on the Coated Strawberry's Total Flavonoid (TF) Amount

Figure 4B compares TF levels in uncoated and coated strawberries kept at 4°C ± 0.5°C. TF progressively decreased in both fruits throughout the storage time, but uncoated fruits showed the greatest declines. Undoubtedly, CMC + G + LEO (3%) and CMC + G + LEO (1.5%) did not significantly vary. Nevertheless, TF declined in the CMC + G + LEO (3%) coating to 3.93 mg CE/100 g. Regarding this, (Khalifa et al. 2016) reports that the decrease in total flavonoid levels in uncoated and coated samples is closely related to the level of antioxidant activity and the respiration rate process. It is observed that the value of flavonoids declined in uncoated and coated treatments with time. On the other hand, the CSM + CNF 0.8% coated strawberries indicate oxygen penetration inhibition, delaying the breakdown of flavonoids substantially. The result of our study could be linked to the protracted fruit from deterioration due to the CMC + G + LEO coating.

### 3.7 The Impact of Coating on the Antioxidant Activity (AOA) of Coated Strawberries

AOA was measured in uncoated and coated strawberries kept at 4°C ± 0.5°C for 16 days. The longer the storage period, the lower the antioxidant activity. The findings are shown in Figure 4C. The AOA of strawberry fruit coating with CMC + G + LEO (1.5% and 3%) displays that it slowly decreased (46.48 μmol TE g^−1^ fw, 41.33 μmol TE g^−1^ fw) during the storage period compared with control, which reduced rapidly from 60.99 to 16.10 μmol TE g^−1^ fw. Also, for CMC + G and CMC + G + LEO (0.5%) was (24.27 μmol TE g^−1^ fw, 34.01 μmol TE g^−1^ fw). Tumbarski et al. (2019) reported that the application of an edible coating effectively delayed the reduction in AOA in strawberries. This effect was attributed to the coating acting as a barrier, which reduced oxygen levels and increased carbon dioxide (CO_2_) concentrations, thereby slowing the respiration rate.

### 3.8 Impact of Coating on Fresh Strawberry Yeast and Mold Counts

**FIGURE 5:**
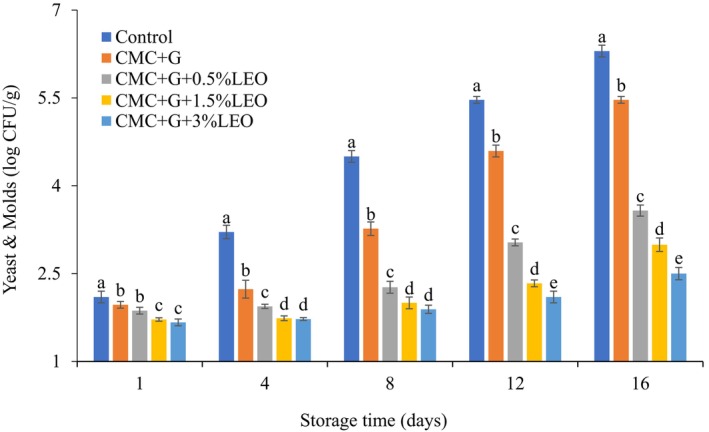
Total counts of yeast and mold (cfu/g) of strawberries.

Figure 5 shows the impact of the various treatments on mold and yeast counts on strawberries for 16 days of storage at 4°C ± 0.5°C. Yeast and mold count of coated CMC + G enrichment with varying concentrations of LEO (0.5%, 1.5%, and 3%) were (2.5, 2.9, and 3.5 log CFU/g) less than samples that were CMC + G coated and uncoated (5.4 and 6.3 log CFU/g). According to Shahbazi (2018), yeast and mold growth on the surface of strawberries was slowed during 12 days of storage using CMC + CH supplemented by *Mentha spicata* essential oil. The effective treatment for mold and yeast was CMC + G + LEO (1.5% and 3%), which reduced growth by (2.9 log CFU/g and 2.5 log CFU/g), respectively. Azarakhsh et al. (2014) findings of this investigation indicated that in all fruits treated with CMC + G enhanced with various concentrations of LEO, the mold and yeast colonies were under acceptable levels for those microorganisms (4 log CFU/g). Dong and Wang (2017) suggest that this result might be explained by a significant crosslinking effect caused by the interaction between the garlic essential oil (GEO) level and CMC. EOs most likely created a rather dense layer that served as a barrier against microbial activity.

### 3.9 Sensory Analysis

**FIGURE 6:**
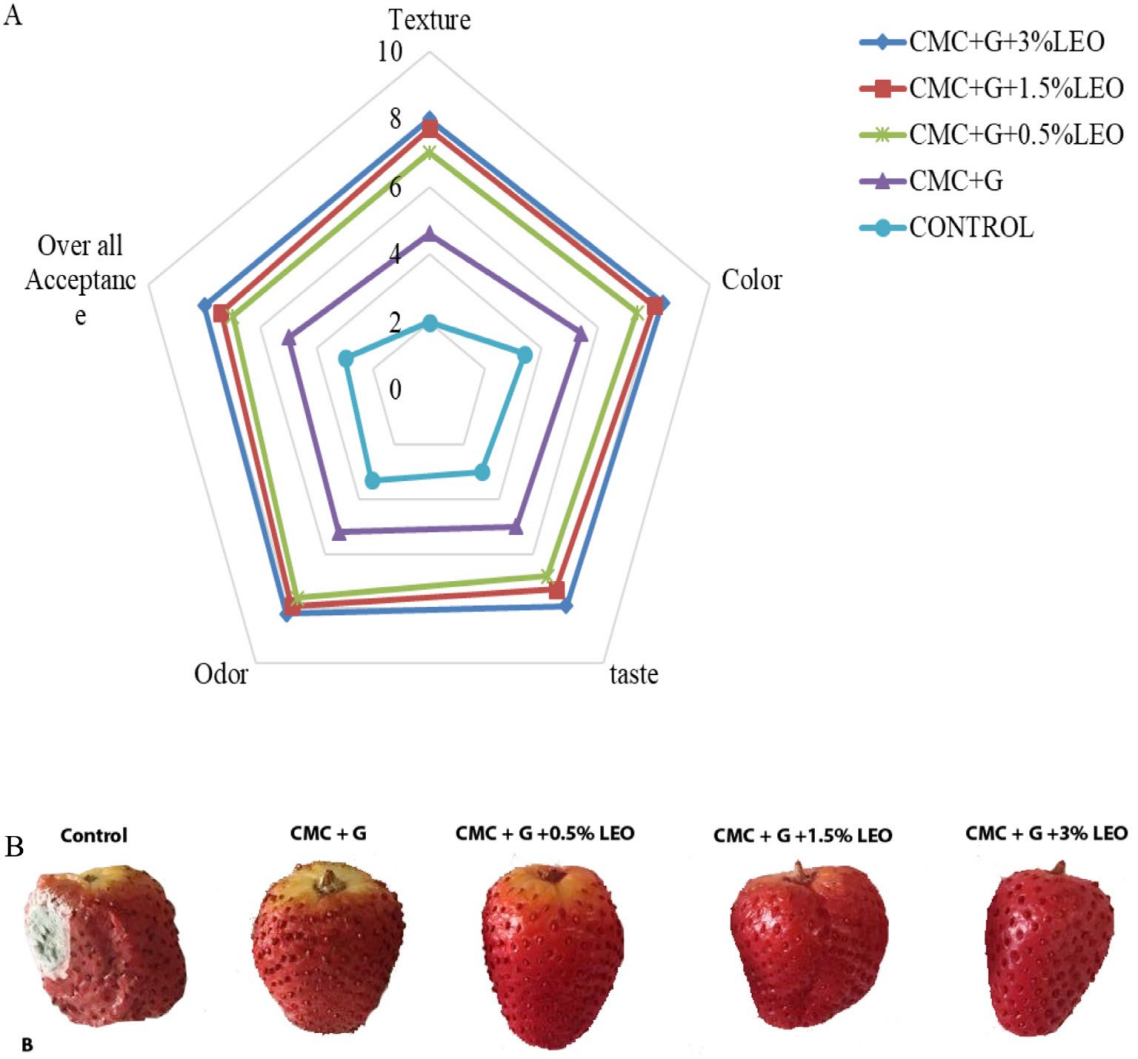
Sensory analysis (A) fresh strawberry coated with CMC + G enriched with LEO after 16 days of storage at (4°C). (B) Control (uncoated) fresh strawberries and fresh strawberries coated by CMC + G and incorporated various concentrations of LEO after 16 days of storage at 4°C.

Figure 6A displays the sensory assessment results of strawberries from all the groups. We compared all the treatments for a 16‐day storage at 4°C ± 0.5°C. Uncoated strawberries were impacted by molds that are not satisfactory for clients, although the quality of CMC + G is higher than uncoated and not as good as CMC + G with varying LEO concentrations. Based on (Dong and Wang 2017) reported that the strawberries coated with CMC in combination with garlic essential oil (2%) maintained the greatest ratings for taste, color, texture, overall acceptability, and flavor over 6 days of storage in contrast to the uncoated strawberries, which deteriorated and displayed an unattractive appearance. Furthermore, in contrast to the uncoated treatment, strawberries coated with CMC + G enriched with varying LEO concentrations (0.5%, 1.5%, and 3%) obtained higher panelist ratings for appearance taste (6.8, 7.3, and 7.9 points, respectively), color (7.4, 8, and 8.3 points, respectively), odor (7.6, 7.9, and 8.2 points, respectively), overall acceptability (7, 7.4, and 8 points, respectively), and texture (7, 7.7, and 8 points, respectively). In addition, it can be inferred that the application of a coating comprising CMC + G in conjunction with LEO significantly enhanced the sensory attributes appearance, taste, color, and overall acceptability of fresh strawberries in comparison with both CMC + G and control samples.

## 4 Conclusion

Coating strawberries with a CMC + G combination with various concentrations of LEO plays a crucial role in preserving antioxidant compounds while simultaneously minimizing weight loss, respiration rate, and microbial proliferation. Doing so effectively slows down deterioration and delays the onset of senescence to 16 days at 4°C ± 0.5°C. Dipping was the primary technique used to apply the edible coating to the surface of the strawberries. Furthermore, the fruits' SSC, TA, and pH levels were maintained. According to our results, formulations based on CMC + G combined with LEO (0.5%, 1.5%, and 3%) had higher preservation effects than CMC + G. The results also observed that strawberries coated with CMC + G containing LEO acted as a barrier to microbial growth. Therefore, formulation coatings containing 3% LEO have better inhibitory properties (2.5 CFU/g) across 16 days of storage. The addition of LEO to the CMC + G coating showed a greater level of acceptability for the color, flavor, and texture than the control and CMC + G. Coating strawberries with CMC + G + LEO (3%) was the most effective method for preserving their nutritional value.

## Author Contributions

**Arkan Mohammed Hassan:** conceptualization (equal), investigation (equal), methodology (equal), supervision (equal), writing – original draft (equal). **Ammar B. Altemimi:** formal analysis (equal), methodology (equal), writing – review and editing (equal). **Babak Ghanbarzadeh:** conceptualization (equal), methodology (equal), resources (equal), validation (equal), writing – review and editing (equal). **Perihan Adun:** data curation (equal), methodology (equal), writing – review and editing (equal). **Khaled Arab:** formal analysis (equal), investigation (equal), software (equal), writing – original draft (equal). **Sonya Ibrahim:** data curation (equal), investigation (equal), writing – original draft (equal). **Farhang Hameed Awlqadr:** data curation (equal), methodology (equal), writing – review and editing (equal). **Mohammad Ali Hesarinejad:** conceptualization (equal), methodology (equal), validation (equal), writing – review and editing (equal). **Tarek Gamal Abedelmaksoud:** data curation (equal), formal analysis (equal), methodology (equal), software (equal), writing – review and editing (equal).

## Consent

Informed consent was obtained from all subjects involved in the study.

## Conflicts of Interest

The authors declare no conflicts of interest.

## Data Availability

Data are contained within the article.
